# Preoperative CD52 Level Predicts Graft Survival following Kidney Transplantation

**DOI:** 10.1155/2022/8949919

**Published:** 2022-09-16

**Authors:** Ali Ramouz, Rajan Nikbakhsh, Elias Khajeh, Mahmoud Sadeghi, Volker Daniel, Paul Schmitzler, Christian Morath, Martin Zeier, Arianeb Mehrabi, Hani Oweira

**Affiliations:** ^1^Department of General Visceral, and Transplantation Surgery, University of Heidelberg, 69120 Heidelberg, Germany; ^2^Transplantation Immunology, University of Heidelberg, 69120 Heidelberg, Germany; ^3^Center for Infectious Disease Virology, University of Heidelberg, 69120 Heidelberg, Germany; ^4^Division of Nephrology, University of Heidelberg, 69120 Heidelberg, Germany; ^5^Department of Surgery Medical Faculty Mannheim, University of Heidelberg, 68167 Mannheim, Germany

## Abstract

Several factors have been reported to affect graft survival following kidney transplantation. CD52 molecules may increase T cell proliferation and activation, which may contribute to acute graft rejection and graft survival. In the current study, we studied the possible value of preoperative CD52 levels in predicting graft survival following renal transplantation. Ninety-six patients with end-stage renal disease who had kidney transplantation were included in the study from our prospective cohort. Blood samples were taken one day before surgery, and plasma CD52 levels were measured using ELISA (Cloud-Clone Corp., Houston, TX, USA). Acute rejection, acute tubular necrosis, delayed graft function, graft loss, BK infection, cytomegalovirus infection, and graft survival were evaluated. The mean age of recipients was 50.08 ± 12.82 years, and 64.6% were male. The incidence of delayed graft function, acute rejection, graft loss (*p* < 0.01), BK virus infection, and serum creatinine levels were significantly higher in recipients with high preoperative CD52 levels six months after transplantation (*p* < 0.05). Kaplan–Meier analysis revealed that three-year graft survival was significantly higher in patients with low preoperative CD52 levels (*p* < 0.0001). Univariate and multivariate Cox regression analyses showed that serum creatinine levels (hazard ratio [HR] = 1.7, *p* < 0.05), acute rejection (HR = 2.919, *p* < 0.05), and preoperative CD52 levels (HR = 3.114, *p* < 0.05) were independent prognostic factors for graft survival after kidney transplantation. We showed that high preoperative CD52 levels are associated with higher rates of acute rejection, delayed graft function, and BK virus infection and lower rates of graft survival after kidney transplantation.

## 1. Introduction

Patients with end-stage renal disease (ESRD) are optimally treated with kidney transplantation (KTx) [1] because it is associated with better quality of life and longer life expectancy [2]. Although patient and graft survival have considerably increased following KTx in recent decades, KTx recipients still have a lower survival rate than healthy individuals because of organ dysfunction and graft loss [3]. Acute graft rejection is one of the main causes of long-term graft loss after KTx, so understanding the pathophysiology of graft rejection is crucial for improving graft survival [4–6].

Recent research has suggested that graft survival can be predicted in KTx recipients by examining immunological markers in peripheral blood before and after KTx [7]. For example, significantly higher numbers of regulatory T cells, CD19+CD24highCD27+B regulatory cells, and CD19+CD24highCD38high transitional B cells were reported in the peripheral blood of tolerant KTx recipients than in recipients with chronic rejection after KTx [8–10]. Another study reported that high preoperative CD200 levels increase the risk of immunosuppression and cytomegalovirus (CMV) infection and that higher preoperative CD200R1/CD200 ratios predict the risk of acute rejection following KTx [11].

CD52 is a nonmodulating cell membrane glycoprotein [12, 13] that is expressed in immune cells such as lymphocytes, monocytes, and dendritic cells [14]. Soluble CD52 binds to B cells and T cells to reduce cell growth and induce apoptosis [15]. CD52-presenting cells eliminate CD4+ and CD8+ T cells via complement- and noncomplement-mediated mechanisms, and the anti-CD52 monoclonal antibody alemtuzumab reduces graft rejection by increasing the number of CD4+ and CD25+ regulatory T cells [15–17].

CD52 promotes the activation and proliferation of T cells [14, 18, 19]. However, the ability of preoperative CD52 levels to predict transplant outcomes and graft survival after KTx has not been evaluated [16]. In this study, we investigated the correlation between preoperative CD52 levels and other risk factors and acute graft rejection and graft survival following KTx.

## 2. Materials and Methods

### 2.1. Study Population

In this prospective cohort, we collected data of 96 consecutive ESRD patients who underwent KTx in our center between January 2015 and December 2017. The study was carried out in accordance with the Helsinki Declaration, and informed consent was obtained from all participants. Study participation did not affect the management or treatment of patients. The study was approved by the University of Heidelberg's ethical committee (S-225/2014). Patients under the age of 18 at the time of transplantation, as well as those who received a combined organ transplant (simultaneous transplantation of kidney and liver, heart, or pancreas), were excluded from the study. Patients who lost their kidney graft within 30 days of receiving KTx were also excluded from the study.

### 2.2. Data Collection and Extraction

Demographic and baseline characteristics were collected for all participants. Patient information was extracted from the university hospital's database. Preoperative factors included serum creatinine, serum CD52, previous transplantation, and human leukocyte antigen (HLA) mismatches. Intraoperative factors included cold ischemia time, and postoperative outcomes comprised acute tubular necrosis, acute allograft rejection, delayed graft function, CMV (re)activation, BK virus infection, and serum creatinine (measured at three, six, and 12 months after transplantation). Preoperative CD52 levels were analyzed in the serum of all recipients to determine whether preoperative CD52 can predict posttransplant events and graft survival. Six postoperative months and one year later, the CMV pp65 antigen levels of patients who received organs from CMV-negative donors were measured. As prophylaxis, patients receiving kidneys from CMV-positive donors were given 900 mg of valganciclovir daily for three months. Following surgery, all patients received conventional treatment with methylprednisolone, mycophenolate mofetil, and a calcineurin inhibitor (cyclosporine or tacrolimus) and were followed up for at least two years.

### 2.3. Obtaining Blood Samples and Measuring CD52 Plasma Levels

On the day before KTx, blood samples were taken from each patient. Plasma was centrifuged at 1.550xg for 10 minutes to separate it from blood cells within two hours of collection and plasma was snap-frozen and stored at −30°C. Plasma CD52 levels were measured using ELISA in accordance with the manufacturer's instructions (Cloud-Clone Corp., Houston, TX, USA).

### 2.4. Detection of Active CMV Infection by CMV pp65 Antigen

CMV pp65 antigen was measured by drawing 8 mL of blood into an EDTA tube and spinning 500,000 leukocytes down onto a slide using a cytospin centrifuge. Cells were fixed and stained with an anti-CMV pp65 mouse monoclonal antibody and then incubated in an antimouse immunoglobulin G FITC-labeled antibody [20]. The number of CMV pp65-positive cells was counted using ultraviolet light microscopy. A positive result was defined as more than three out of 500,000 CMV pp65-positive cells [21].

### 2.5. Detection of Active BK Virus Infection by Real-Time PCR

DNA was extracted from 200 *μ*L of untreated plasma using the QIAamp kit according to the manufacturer's instructions (Qiagen, Hilden, Germany). The main T-antigen in the BK virus genome was quantified from 5 mL of extracted DNA using TaqMan real-time PCR [22]. The detection limit was set at 50 copies/mL. A BK viral load of more than 10,000 copies/mL was considered an active infection [23].

### 2.6. Quantifying Posttransplant Outcomes

Acute transplant rejection was determined by the presence of necrotic renal tubules in renal biopsies [24]. Delayed graft function was determined by the temporary requirement for one or more dialysis treatments during the first postoperative week. Graft loss was determined by the need for retransplantation or permanent dialysis during follow-up.

### 2.7. Statistical Analysis

Statistical analysis was conducted utilizing IBM SPSS Statistics for Windows, version 27.0 (IBM Corp. Released 2013. Armonk, NY) and GraphPad Prism version 9. Continuous variables were presented as mean and standard deviation, and categorical data were shown as percentages. Normal distribution of the data was determined by the Shapiro–Wilk test. For normally distributed variables, unpaired *t* tests were used to analyze continuous variables and the chi-squared test was used to analyze categorical variables. Variables that were not normally distributed were analyzed by Mann–Whitney *U* and Fisher's exact nonparametric tests. Receiver operating characteristic (ROC) curves were generated by plotting sensitivity against 1 − specificity. The optimal cutoff values for ROC curves and area under the curves (AUCs) were calculated using the Youden index (YI = sensitivity + specificity 1). The effect of different factors on graft survival was assessed using Kaplan–Meier curves. In the Kaplan–Meier curve analysis, differences between subgroups were determined using a log-rank test. Univariate and multivariate analyses were used in the Cox regression model to examine the prognostic value of the factors, and the results were presented as hazard ratios (HRs) with 95% confidence intervals (CIs). Statistical significance was defined as a *p* value < 0.05.

## 3. Results

### 3.1. Patient Characteristics

The demographic and clinicopathologic characteristics of KTx recipients are shown in Table 1. The average age of recipients was 50.08 ± 12.82 years, and 64.6% of recipients were male. Glomerulonephritis was the most common etiology of ESRD (53 recipients; 55.2%), followed by autoimmune and polycystic kidney diseases (21 recipients; 21.9%). Almost all recipients (96.9%) had previously undergone dialysis, with a mean duration of 65.9 ± 53.6 months. A repeat KTx was performed in 14.6% of recipients. The preoperative CD52 plasma level was 317.7 ± 220.5 pg/mL, and the preoperative serum creatinine level was 7.42 ± 2.54 mg/dl. CMV reactivation was found in 13 (13.5%) patients and BK virus infection in 17 (17.7%) patients. Delayed graft function was observed in 20 patients (20.8%), acute allograft rejection in 17 patients (17.7%), and acute tubular necrosis in three patients (3.1%) after KTx. Finally, graft loss was observed in 22 recipients (22.9%) following KTx (Table 1).

### 3.2. Graft Survival and Serum CD52 Levels

**Figure 1:**
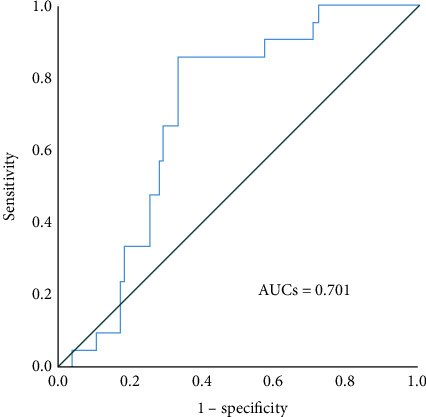
ROC curve analysis reveals an AUC value of 0.701 for preoperative CD52 levels to differentiate between patients with and without graft loss after KTx.

**Figure 2:**
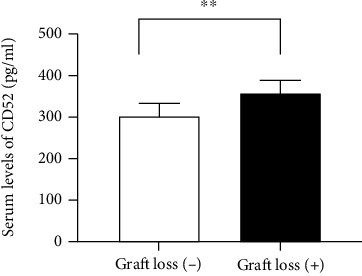
Comparison of preoperative serum CD52 levels in recipients with and without graft loss. Values are expressed as the mean ± SEM. *n* = 22 patients in the graft loss (+) group and *n* = 74 patients in the graft loss (−) group were analyzed using Mann–Whitney test. ∗∗*p* < 0.01.

Recipients were divided into a graft loss group and no graft loss group, and the preoperative CD52 levels were compared between these two groups. The Mann–Whitney *U* test showed that recipients with graft loss had significantly greater CD52 levels than recipients without graft loss did (*p* < 0.01, Figure 1). A cutoff value of 260 pg/mL was determined as the optimal CD52 level for predicting postoperative graft loss using a time-dependent ROC curve. Based on this cutoff value, we stratified patients into two groups: a low CD52 group (<260 pg/mL, *n* = 57) and a high CD52 group (≥260 pg/mL, *n* = 39). The AUC for CD52 serum levels was 0.701 (95% CI: 0.593–0.809, *p* = 0.005, Figure 2). The mean age of patients was significantly higher in the high CD52 group than in the low CD52 group (52.73 ± 11.71 vs 46.91 ± 13.7, *p* < 0.05). The etiology of ESRD was not significantly different between the low CD52 and high CD52 groups. Serum creatinine levels were significantly higher in the high CD52 group six months after KTx (*p* < 0.05).

**Figure 3:**
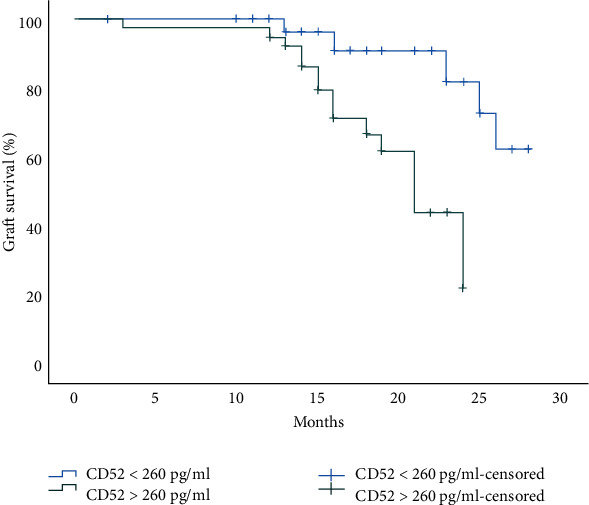
Kaplan–Meier curve stratified by preoperative serum CD52 levels in recipients undergoing KTx.

We also compared postoperative outcomes between the high CD52 and low CD52 groups. The rate of delayed graft function was significantly higher in the high CD52 group than in the low CD52 group (14/20, 35.9% vs 6/20, 10.5%; *p* < 0.01). The rate of acute rejection was also higher in the high CD52 group (*p* < 0.01), and BK virus infection occurred more frequently in the high CD52 group (28.2%) than in the low CD52 group (10.5%) (*p* < 0.05), whereas no differences were observed in the rate of CMV infection between the two groups. The graft loss rate was also significantly higher in the high CD52 group (*p* < 0.01) (Table 1). Kaplan–Meier analysis showed better graft survival in the low CD52 group than in the high CD52 group (*p* < 0.0001, Figure 3).

### 3.3. Preoperative Level of CD52 Can Predict Graft Survival following Renal Transplantation

Cox regression analysis was carried out to determine whether the examined factors could predict graft survival following KTx. In univariate analyses, six-month postoperative serum creatinine, acute allograft rejection, delayed graft function, BK virus infection, and CD52 plasma levels were found to predict graft loss after KTx (Table 2). Interestingly, in multivariate analysis, only serum CD52 levels, six-month creatinine levels, and acute rejection were found to be independent prognostic factors of graft survival (Table 2).

## 4. Discussion

It has been shown that alemtuzumab, an anti-CD52 antibody, can induce lymphocyte depletion [14, 25]. Both T cells and B cells could eventually reconstitute in the peripheral blood, resulting in the recovery of their initial population. Reconstitution of T cells and B cells may shift the immune system toward an anti-inflammatory pattern, which may be associated with a more stable and effective graft function [26]. However, the ability of preoperative CD52 levels to predict KTx outcomes such as rate of acute rejection and graft survival has not been investigated [14]. We measured preoperative CD52 serum levels in patients undergoing KTx and assessed the effect of these serum levels and other preoperative, intraoperative, and postoperative factors on surgical outcomes and graft survival. We found that acute rejection and post-transplant serum creatinine six months after KTx and preoperative serum CD52 levels predicted graft survival following KTx. Interestingly, BK virus infection, acute allograft rejection, delayed graft function, and graft loss were more frequent in recipients with higher levels of preoperative CD52.

Our findings that high preoperative CD52 levels can predict poor KTx outcomes supports findings that the anti-CD52 antibody alemtuzumab can reduce acute graft rejection and improve survival outcomes after KTx [25]. Numerous studies have shown that alemtuzumab improves survival and reduces rejection in recipients at high risk for graft rejection. These high-risk patients include older patients, patients with more than two HLA mismatches, and patients with a history of transplantation [16]. Alemtuzumab improves KTx outcomes by modulating CD4 and CD8 expression on the T cell surface and by expanding CD4+CD25+Foxp3+ regulatory T cells [18, 27, 28]. It was recently shown that successive targeting of CD52 and TNF-*α* minimized early immunosuppressive therapy following KTx [18]. In this study, we showed that higher preoperative CD52 levels are associated with delayed graft function, suggesting that CD52 levels can be measured before KTx to predict graft outcomes. We also found that acute graft rejection predicts poor graft survival after KTx. Another study demonstrated that HLA mismatches, delayed graft function, and acute graft function were associated with lower graft survival and that higher levels of CD52 could activate CD4+ and CD8+ T cells to increase graft rejection [14]. Another study showed that 20% of regulatory T cells were CD4+CD25+FoxP3+ after alemtuzumab treatment compared with 4% of cells in recipients not treated with the antibody and 3% of cells in healthy controls. This indicates that alemtuzumab reduces graft rejection by increasing CD4, CD25, and FoxP3 expression in regulatory T cells [29–31].

Because CD52 regulates immune cells and the anti-CD52 antibody alemtuzumab reduces immunosuppression after transplantation, we hypothesized that increased CD52 serum levels might be linked to a higher incidence of infection after transplantation. Indeed, we found that the rate of BK virus infection was higher in patients with high preoperative CD52 levels. In agreement with our findings, other studies have shown higher rates of posttransplant infection in patients with immunosuppression. Epstein–Barr virus infection was strongly associated with T cell depletion and was predicted by cell-mediated immunity and hematological parameters [32, 33]. Similarly, Fernández-Ruiz et al. reported that high CD30 serum levels considerably increased the rate of infection following KTx [34].

Infection with the BK virus was recently shown to decrease graft survival and increase the rate of acute allograft rejection after KTx [35]. In present study, patients with BK viremia had also higher level of preoperative CD52. It can be hypothesized that higher level of CD52 might reflect the highly immunologically active milieu in patients with acute rejection. These patients receive more intensive types and dosage of immunosuppressive agents in response to the acute rejection. Considering the higher risk of BK viremia in association with intense immunosuppression, particularly alemtuzumab induction, the higher rate of BK viremia might actually be related to increased immunosuppression resulting from the treatment of the more frequent acute rejection episodes in these recipients, rather than a direct effect of high pretransplant CD52 level [20, 22].

There are some limitations to this prospective cohort investigation. The patient population was heterogenous, and there was no control group with comparable features. In addition, the sample size was small. Therefore, further studies with larger sample sizes are required to confirm whether preoperative serum CD52 levels can predict graft rejection and survival in the long term.

## 5. Conclusions

We found that acute allograft rejection, serum creatinine levels six months after KTx, and serum CD52 levels before KTx are independent prognostic factors affecting surgical outcomes and graft survival. We showed for the first time that higher serum CD52 levels are associated with higher rates of acute rejection, delayed graft function, and BK virus infection.

## Figures and Tables

**Table 1:** Baseline characteristics, posttransplant outcomes, and clinical characteristics of patients undergoing KTx.

Variables	Total (*n* = 96)	CD52 <260 pg/mL (*n* = 57)	CD52 ≥260 pg/mL (*n* = 39)	*p* value
Baseline and clinical characteristics				
Age (years)	50.08 ± 12.82	46.91 ± 13.7	52.73 ± 11.71	**<0.05**
Sex (female/male)	34 (35.4%)/62 (64.6%)	21 (36.8%)/36 (63.2%)	13 (66.7%)/26 (33.3%)	NS
Indication for transplantation				
Glomerulonephritis (*n*, %)	53 (55.2%)	31 (54.4%)	22 (56.4%)	NS
Autoimmune/polycystic disease (*n*, %)	21 (21.9%)	15 (26.3%)	6 (15.4%)	NS
Pyelonephritis (*n*, %)	7 (7.3%)	5 (8.8%)	2 (5.1%)	NS
Diabetic nephropathy (*n*, %)	6 (6.3%)	3 (5.3%)	3 (7.7%)	NS
Hypertensive nephropathy (*n*, %)	1 (1%)	0 (0%)	1 (2.6%)	NS
Other/unknown (*n*, %)	8 (8.3%)	3 (5.3%)	5 (12.8%)	NS
Preoperative dialysis				
No dialysis	3 (3.1%)	2 (3.5%)	1 (2.6%)	
Hemodialysis (*n*, %)	85 (88.5%)	52 (91.2%)	33 (84.6%)	NS
Peritoneal dialysis (*n*, %)	8 (8.3%)	3 (5.3%)	5 (12.8%)	NS
Duration of dialysis (months)	65.9 ± 53.6	63.7 ± 53.3	71.8 ± 55.3	NS
Number of total HLA mismatch (*n*, %)				
≤2	34 (35.4%)	22 (38.6%)	12 (30.8%)	NS
>2	62 (64.6%)	35 (61.4%)	27 (69.2%)	NS
Preoperative plasma level of CD52 (pg/mL)	317.7 ± 220.5	294.59 ± 189.9	334.88 ± 243.4	**<0.0001**
Cold ischemia time (minutes)	569.4 ± 401.3	573.1 ± 395.3	579.2 ± 416.9	NS
Repeated KTx (*n*, %)	14 (14.6%)	7 (12.3%)	7 (17.9%)	NS
Serum creatinine				
Pretransplantation	7.42 ± 2.54	7.22 ± 2.44	7.47 ± 2.65	
3 months after transplantation	1.67 ± 0.53	1.62 ± 0.53	1.74 ± 0.56	NS
6 months after transplantation	1.77 ± 0.8	1.65 ± 0.78	1.94 ± 0.85	**<0.05**
12 months after transplantation	1.71 ± 0.9	1.65 ± 0.88	1.85 ± 0.98	NS
Postoperative factors and outcomes				
Acute allograft rejection (*n*, %)	17 (17.7%)	5 (8.8%)	12 (30.8%)	**<0.01**
Acute tubular necrosis (*n*, %)	3 (3.2%)	2 (3.6%)	1 (2.6%)	NS
Delayed graft function (*n*, %)	20 (20.8%)	6 (10.5%)	14 (35.9%)	**<0.01**
CMV (re)activation (*n*, %)	13 (13.5%)	8 (14%)	5 (12.8%)	NS
BK virus infection	17 (17.7%)	6 (10.5%)	11 (28.2%)	**<0.05**
Graft loss (*n*, %)	22 (22.9%)	8 (14%)	14 (35.9%)	**<0.01**

HLA: human leukocyte antigen; CMV: cytomegalovirus; KTx: kidney transplantation.

**Table 2:** Univariate and multivariate analyses of preoperative and postoperative factors affecting graft loss.

Variables	Comparison	*n* (%)	Univariate		Multivariate	
			HR (95% CI)	*p* value	HR (95% CI)	*p* value
Age	≥50	56 (58%)	1.406 (0.582–3.395)	0.449		
	<50	40 (42%)				

Gender	Male	62 (64.5%)	0.849 (0.346–2.085)	0.721		
	Female	34 (35.5%)				

Serum creatinine (6 months after KTx)	0.7–6		1.908 (1.192–3.053)	**0.007**	1.700 (1.022–2.828)	**0.041**

Acute allograft rejection	Yes	17 (17.7%)	2.997 (1.239–7.250)	**0.015**	2.919 (1.118–7.623)	**0.029**
	No	79 (82.3%)				

DGF	Yes	20 (20.8%)	2.672 (1.113–6.412)	**0.028**	1.361 (0.500–3.706)	0.546
	No	76 (79.2%)				

Total HLA mismatch	≤2	34 (35.4%)	1.169 (0.868–1.576)	0.304		
	>2	62 (64.6%)				

BK virus infection	Yes	17 (17.7%)	2.673 (0.856–8.348)	**0.091**	0.988 (0.224–4.366)	0.987
	No	79 (82.3%)				

Serum levels of CD52	CD52 ≥260 pg/mL	39 (40.6%)	3.168 (1.346–7.454)	**0.008**	3.114 (1.026–9.604)	**0.045**
	CD52 <260 pg/mL	57 (59.4%)				

DGF: delayed graft function; HLA: human leukocyte antigen; KTx: kidney transplantation.

## Data Availability

The data used to support the findings of this study are available from corresponding author upon request.
